# Thymoma-Associated Myasthenia Gravis With Myocarditis

**DOI:** 10.7759/cureus.42473

**Published:** 2023-07-26

**Authors:** Shihab Sarwar, Oyebimbola Oyewunmi, Karundat Bhola, Bobak Heydari

**Affiliations:** 1 Division of Cardiology, University of Ottawa Heart Institute, University of Ottawa, Ottawa, CAN; 2 Department of Medicine, Cumming School of Medicine, University of Calgary, Calgary, CAN; 3 Department of Radiology, Stephenson Cardiovascular Imaging Centre, University of Calgary, Calgary, CAN; 4 Department of Cardiac Sciences, Stephenson Cardiovascular Imaging Centre, University of Calgary, Calgary, CAN

**Keywords:** autoimmune, mri cardiac, thymoma, myocarditis, myasthenia gravis

## Abstract

Myasthenia gravis (MG) complicated by myocarditis is a rare autoimmune manifestation. We present a patient who initially presented with a suspected ST-segment elevation myocardial infarction (STEMI) with angiographically normal coronary arteries. A chest CT scan revealed a large homogenous soft-tissue density anterior mediastinal mass suspicious of thymoma. Neurological deterioration in the hospital suggested a diagnosis of MG with subsequent electromyography and nerve conduction studies (EMG/NCS) and repetitive nerve stimulation (RNS) confirmation. A cardiac magnetic resonance imaging study (CMR) demonstrated diffuse myocardial edema and severe left ventricular (LV) dysfunction and sub-epicardial late gadolinium enhancement (LGE) involving all basal and mid-LV segments in addition to apical inferior and lateral segments. A diagnosis of thymoma-associated MG with myocarditis was made and the patient was successfully treated with immunosuppression. This case highlights the association of myocarditis with MG as a potential complication that should be considered in patients with cardiac symptoms, ECG changes, or biomarker elevation.

## Introduction

Myasthenia gravis (MG) is an autoimmune disease that is mediated by autoantibodies against the acetylcholine receptor and is characterized by fluctuating muscular weakness involving ocular, bulbar, and, less frequently, nuchal or proximal limb muscles [1]. The condition is commonly associated with thymic hyperplasia or thymoma, and less commonly with other conditions such as rheumatoid arthritis and systemic lupus erythematosus [2]. In some cases, MG can also be associated with myocarditis and can cause severe left ventricular (LV) dysfunction and lethal arrhythmia, amongst other complications [3]. While there have been few case reports of thymoma-associated MG with myocarditis, the pathogenesis and clinical features of this condition remain poorly understood. In this report, we present a case of a male patient with thymoma-associated MG and myocarditis, detailing the clinical presentation, diagnostic workup, and management.

## Case presentation

**Figure 1:**
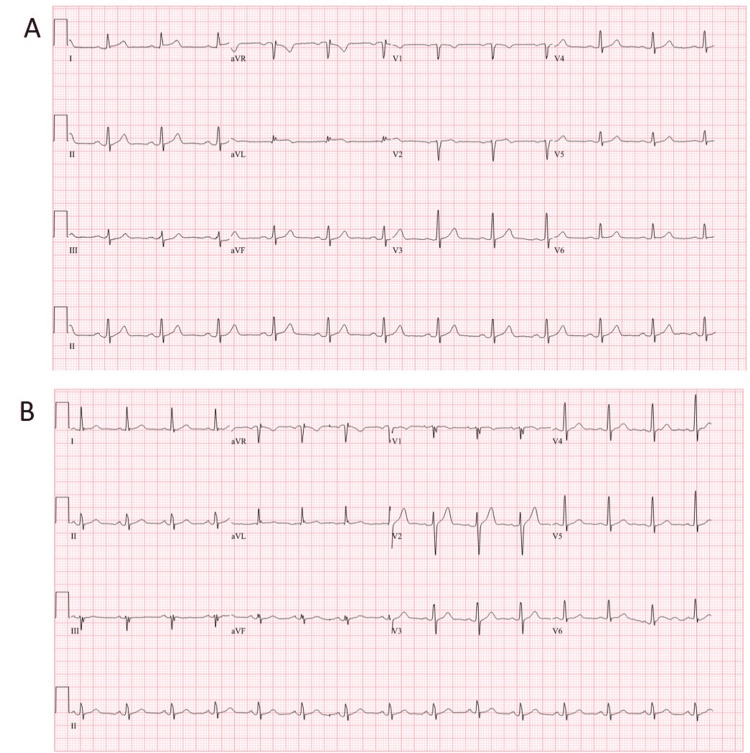
Electrocardiograms on initial presentation and at 8 months from initial hospitalization. A: Initial ECG on presentation to the emergency department demonstrating subtle ST-segment elevation in the high lateral leads I and aVL; B: ECG at 8 months after initial hospitalization shows normal sinus rhythm with fragmented QRS complexes in the inferior leads.

A previously healthy 54-year-old male presented to his primary care provider after 5 days of constant chest heaviness and tightness associated with weakness of his eyelids and arms. He was directed to the emergency department and found to have a normal physical examination despite ongoing chest discomfort. Initial ECG revealed new ST-segment elevations in the lateral limb leads with associated elevation in high-sensitivity troponin T (hsTnT) to 2970 ng/L (the normal, less than 14 ng/L; Figure 1). A diagnosis of suspected ST-segment elevation myocardial infarction (STEMI) was made; the patient was given a ticagrelor and aspirin loading dose, heparin bolus with infusion, and the cardiac catheterization laboratory was activated for primary percutaneous coronary intervention. Invasive coronary angiography revealed no-obstructive coronary artery disease with the left ventriculogram showing no obvious LV dysfunction and left ventricular end-diastolic pressure (LVEDP) measured at 4 mmHg.

**Figure 2:**
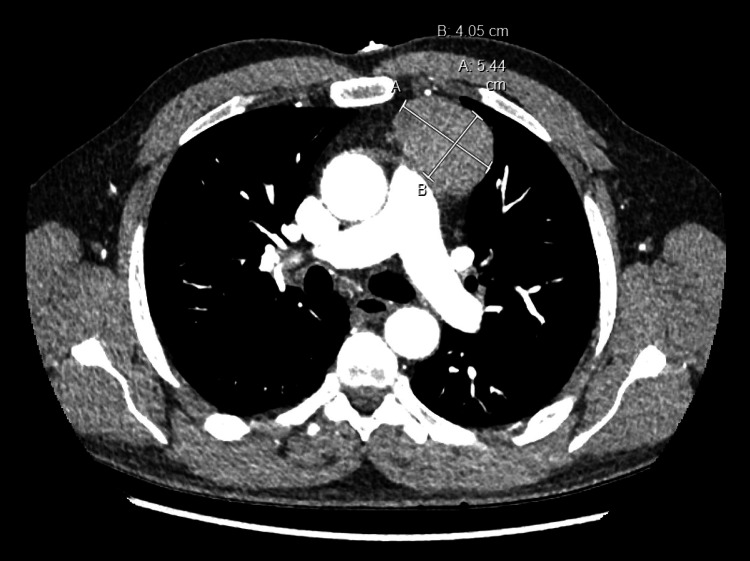
CT chest, abdomen, and pelvis angiography at initial presentation. A homogeneous soft tissue density anterior mediastinal mass, measuring 5.44 cm x 4.05 cm x 4.2 cm (not shown in the figure) is demonstrated on the axial slice.

An initial differential diagnosis for his presentation included myocarditis, myocardial infarction with non-obstructive coronary arteries (MINOCA), and aortic dissection. A thoracic CT angiogram ruled out aortic dissection but demonstrated a large, uniform, soft-tissue mass in the anterior mediastinum measuring 5.44 cm x 4.05 cm x 4.2 cm, likely of thymic origin with differential including lymphoma, thyroid goiter/mass, and teratoma (Figure 2). The viral panel was negative for COVID-19, influenza A/B, respiratory syncytial virus (RSV), and a respiratory viral panel. Serologies for HIV, hepatitis B, and C were unremarkable. Thyroid stimulating hormone level was normal. ANA (1:80, speckled pattern) and ENA (SS-A60 IgG) tests were both positive.

The patient developed increasing left facial heaviness, dysarthria, and oropharyngeal dysphagia over the next few days of hospitalization. An initial contrast-enhanced CT of the brain was unremarkable, while the neurologic examination was notable for bilateral upper eyelid ptosis, prolonged up-gaze with Cogan’s lid twitch sign, weak orbicularis oculi function, and normal strength testing in the limbs, head, and neck. These clinical findings were consistent with neuromuscular junction dysfunction with the leading differential diagnosis of MG (particularly in the setting of suspected thymoma). Nerve conduction studies (NCS) and electromyogram (EMG) were normal, while repetitive nerve stimulation (RNS) testing showed left trapezius and right nasalis with significant decrement. A diagnosis of thymoma-associated MG was made with suspicion of associated myocarditis.

**Figure 3:**
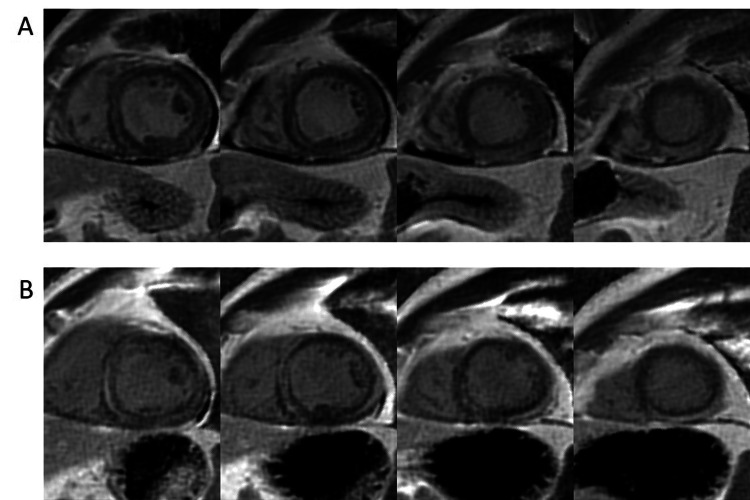
CMR imaging - PSIR short-axis sequence with late gadolinium enhancement. A: Initial presentation; B: Three months follow-up imaging. Follow-up imaging shows persistent subepicardial inflammation, with an overall reduction in the burden of myocardial edema. CMR: Cardiac magnetic resonance PSIR: Phase-sensitive inversion recovery

**Figure 4:**
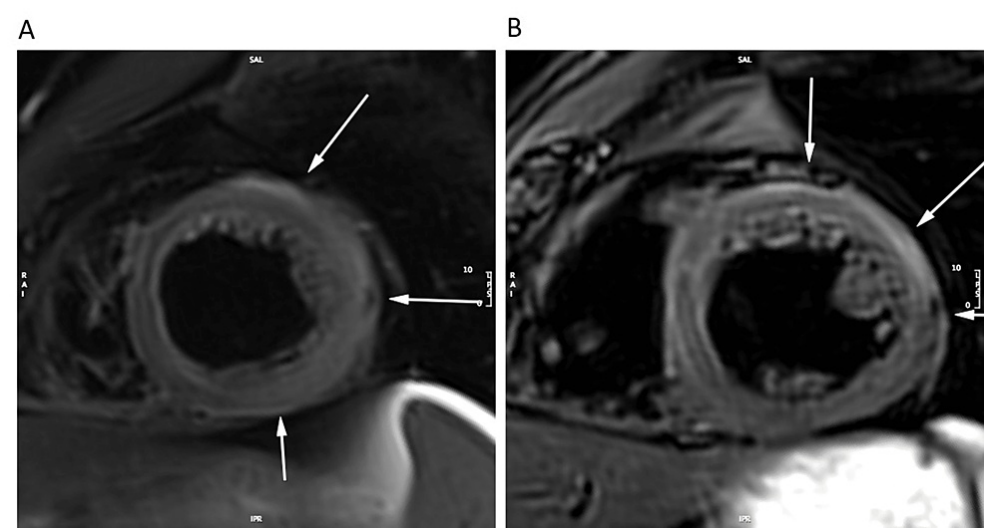
Mid-left ventricle SPAIR on CMR imaging. A: Initial CMR at presentation; B: Follow-up CMR at 3 months after initial imaging. CMR: Cardiac magnetic resonance SPAIR: Spectral attenuated inversion recovery

hsTnT level continued to rise to a peak level of 3507 ng/L with an NT-proBNP level of 763 ng/L. A cardiac magnetic resonance imaging study (CMR) was conducted that demonstrated mildly reduced global left ventricular systolic function (LVEF, 52%) with hypokinetic mid to apical inferior and inferolateral segments; the presence of sub-epicardial late gadolinium enhancement (LGE) involving all basal and mid-LV segments in addition to apical inferior and lateral segments; mid-wall septal LGE; and extensive diffuse myocardial edema by water sensitive imaging (Figure 3A, 4A).

A PET-CT scan was also conducted that demonstrated mild-moderate increased metabolic activity within the thymic mass, but no increased uptake within lymph nodes. Pulse steroids were initiated with methylprednisolone 1000 mg IV daily and intravenous immunoglobulin (IVIG) 1 g/kg daily initially for management of his MG and empiric therapy for possible giant-cell myocarditis prior to cardiac biopsy. His clinical status began to deteriorate with the progression of his bulbar symptoms and the deterioration of his slow vital capacities. Plasmapheresis was initiated, IVIG stopped, and the patient was intubated for type 2 respiratory failure. A right ventricular biopsy was subsequently arranged, with biopsy findings revealing mild edema, a small focus of rare lymphocytes without myocyte damage, mild myocyte hypertrophy, and mild interstitial fibrosis. A diagnosis of thymoma-associated MG with myocarditis was made based on overall clinical presentation and CMR.

The patient demonstrated a therapeutic response after 5 days of methylprednisolone and plasmapheresis. He was subsequently extubated and started on a taper of prednisone 1 mg/kg. The patient was discharged with an outpatient transthoracic ECG one week later that demonstrated normal LV function without regional wall motion abnormalities. Serology for MG returned after several weeks showing positive acetylcholine receptor antibodies.

One month post-discharge, the patient suffered a myasthenic crisis precipitated by COVID-19 infection necessitating ICU admission and plasmapheresis. The patient responded and was maintained on biweekly plasmapheresis, rituximab infusions, prednisone, and pyridostigmine. Thymectomy was arranged with pathology showing thymoma, type B2, Masoka-Koga Stage IIA. An attempt was made post-thymectomy to wean off regular plasmapheresis; however, his symptoms recurred and necessitated the maintenance of biweekly plasmapheresis. Mycophenolate mofetil was added as an additional steroid-sparing immunosuppressant while tapering steroids. A repeat CMR conducted 3 months after the initial presentation demonstrated interval improvement of LVEF (63%) with a reduction in the extent of myocardial edema and sub-epicardial LGE (Figure 3B, 4B). The patient continued to be asymptomatic from a cardiac perspective.

## Discussion

Myocarditis can be an extra neurologic autoimmune manifestation of MG and may present as fulminant myocarditis and cardiogenic shock that is responsive to high-dose steroids and immunosuppression [4]. MG affects primarily skeletal muscle and the pathophysiology of myocardium involvement is unclear but may include either autoimmune or paraneoplastic mechanisms in cases associated with thymoma. From an etiology standpoint, it is suspected that striational antibodies found in MG patients that react with titin, ryanodine receptor (RyR), and Kv1.4 voltage-gated potassium channel may play a role in the pathogenesis of myocarditis in MG patients, or serve as a potential biomarker of cardiac involvement [5,6]. The incidence of thymoma in MG complicated by myocarditis is estimated at 65% [5]. Cardiac biopsies in these patients typically show one or more features of multinucleated giant cells, lymphocytes, myocardial degeneration, necrosis, and fibrosis [5]. Giant-cell myocarditis can also be associated with MG and represents a more serious phenotype of MG myocarditis, with older patients and larger thymomas more likely to develop giant-cell myocarditis [5,7]. The timing of acute myocarditis in relation to the development of MG ranges from 0 days to 17 years [5]. In our patient, cardiac biopsy samples taken did not reveal overt myocarditis, likely due to sampling error. The lack of biopsy-proven findings of myocarditis on histopathology does not exclude the diagnosis, particularly in light of the clinical presentation and imaging findings consistent with myocardial edema/inflammation. Moreover, right ventricular endomyocardial biopsy has poor sensitivity for the histopathologic diagnosis of myocarditis (22.1-28.0% for each individual biopsy sample) [8].

Treatment of MG complicated by myocarditis specifically is not elucidated in current treatment guidelines; however, case reports document response with high-dose glucocorticoids often in combination with other immunomodulatory drugs, including azathioprine, cyclosporine, mycophenolate mofetil, methotrexate, or tacrolimus [5]. IVIG and plasma exchange can also be used in severe cases of MG, while thymectomy in thymoma-associated MG is mainstay of treatment when the clinical status is stable [5]. Despite treatment, a review of 35 patients with MG associated with myocarditis found that in-hospital mortality was 51% [5].

## Conclusions

We present a rare case of a patient with suspected STEMI found to have a thymoma-associated MG complicated by acute myocarditis. Myocarditis is a reported complication of myasthenia gravis and high-index of suspicion should be exercised for this complication in patients diagnosed with MG given the potential for early and late morbidity/mortality. CMR is particularly well-suited to non-invasively evaluate the etiology of unexplained myocardial injury in myasthenia gravis without the use of iodinated contrast or iatrogenic radiation. Further research is needed to better understand the mechanisms underlying myocarditis associated with MG and to develop more effective treatment strategies.
